# Binding for life: corticosteroid binding globulin from vertebrate physiology to human diseases

**DOI:** 10.3389/fendo.2025.1647096

**Published:** 2025-10-16

**Authors:** Philippe Le Rouzic, Karine Rousseau

**Affiliations:** ^1^ Sorbonne Université, Institut National de la Santé et de la Recherche Médicale (INSERM), Centre de Recherche Saint-Antoine (CRSA), Paris, France; ^2^ Muséum National d’Histoire Naturelle, Laboratoire, Physiologie Moléculaire et Adaptation (PhyMA), Paris, France

**Keywords:** corticosteroid binding globulin (CBG), vertebrates, expression, regulation, human diseases

## Abstract

The hypothalamic-pituitary-adrenal/interrenal axis (HPA/HPI) is the neuroendocrine axis which allows vertebrates to cope with changing environments *via* adaptative stress responses. Glucocorticoids (GC) are the main effectors of this corticotropic axis, and their plasma levels (free form) are elevated under stress conditions. In contrast, in normal conditions, in order to prevent their deleterious impact on tissues, GC are found bound to a binding protein, the corticosteroid binding globulin (CBG). This protein, also called transcortin, was discovered in the 1950s, and later shown to be part of the SERPIN family (SERPINA6). Most vertebrates present high levels of bound GC, but some exceptions exist such as lamprey, flying squirrel or New World monkey. In birds, CBG is reported to be the substitute for sex hormone-binding globulin (SHBG) as well since they lack *shbg* gene. In amphibians, CBG binds GC and sex steroids with equivalty -50ently high affinity. In teleosts, up to now, no CBG has been characterized. Mainly synthetized by the liver, the CBG is released in the blood where it serves as a GC transporter and address them to the sites of inflammation and infection. Evidences accumulate to propose CBG as also a GC reservoir. Other functions, under-characterized for the moment, have also been reported for the CBG: extrahepatic CBG could prevent the GC to bind to their receptor(s) intracellularly, and circulating CBG-GC complex could bind to a receptor which allows its internalization in target cells by endocytosis. The concentrations of the plasma CBG show natural physiological variations during specific life-history stages such as during pregnancy and hibernation in mammals, or breeding season in birds. Vertebrates may also present fluctuating CBG when experiencing extreme conditions leading to food deprivation for example. CBG knockout in mice and mutations in human stressed out the functional importance of CBG. In human, a CBG deficit is associated with a number of patho-physiologies including endocrine diseases (hypo- or hyper-thyroidism, obesity) and pro-inflammatory pathologies (sepsis, burning). Our review begins by a description of CBG discovery, characterization and measurement in vertebrates. A focus on the variations of CBG concentrations in various physiological conditions or under non-natural situations in vertebrates follows. The current knowledge on the different functions reported for CBG is then unfold. Our review ends with CBG pathological alterations observed in human to evidence how this protein could have therapeutic uses.

## 1 Introduction

**Figure 1:**
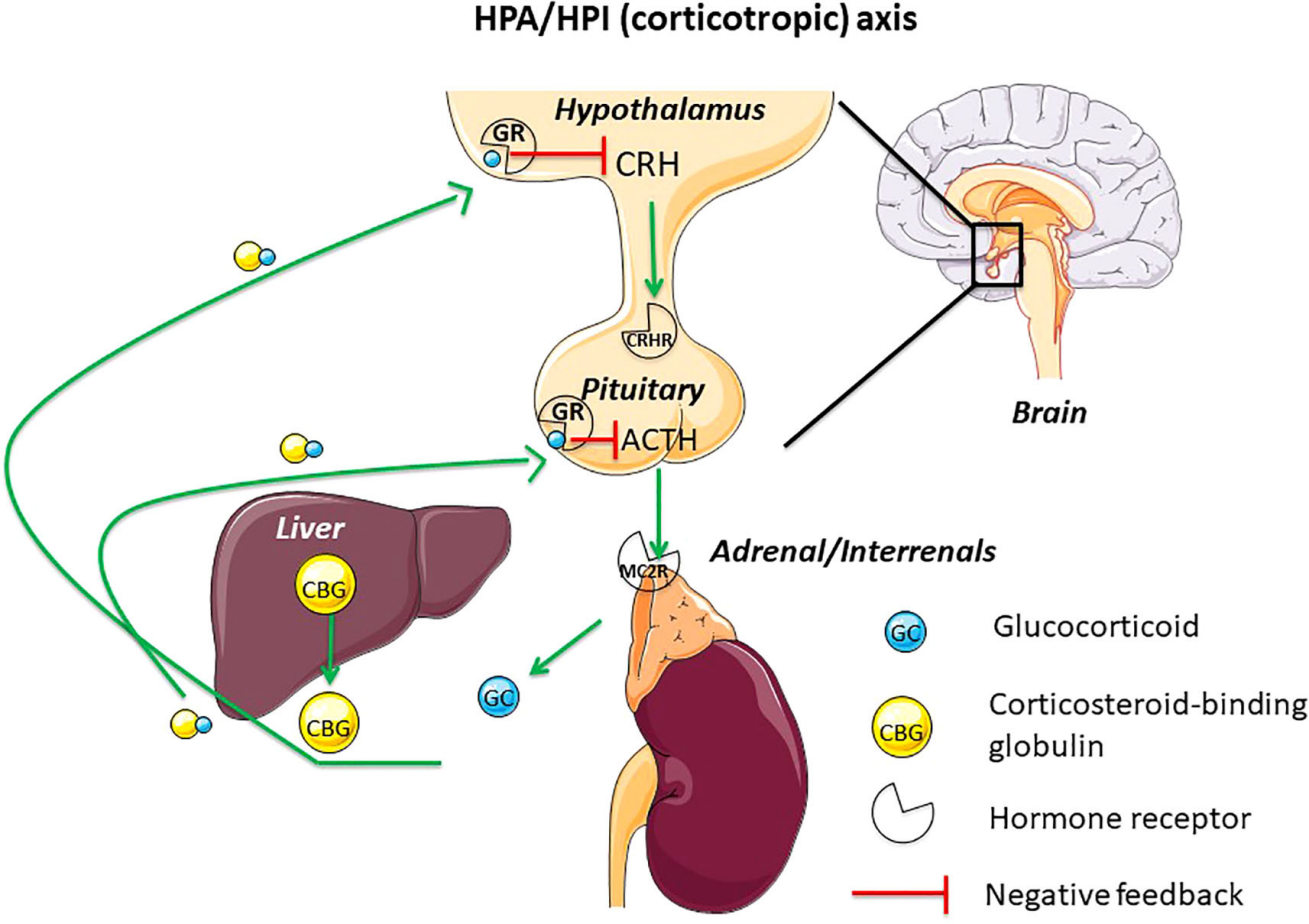
This figure displays the main actors of the hypothalamus-pituitary-adrenal/interrenal axis (HPA/HPI) (corticotropic or stress axis) in vertebrates. CBG, mainly synthetized by the liver, is released in the blood where it binds GC. GC will operate subsequently a negative feedback on hypothalamus and pituitary. ACTH, adrenocorticotropic hormone; CBG, corticosteroid-binding globulin; CRH, corticotropin-releasing hormone; CRH-R, corticotropin-releasing hormone receptor; GC, glucocorticoid; GR, glucocorticoid receptor; HPA, hypothalamus-pituitary-adrenal axis; HPI, hypothalamus-pituitary-interrenal axis; MC2R, melanocortin receptor 2.

The neuroendocrine axis which produces glucocorticoids (GC) is commonly named corticotropic axis or hypothalamic-pituitary-adrenal axis (HPA), in mammals and sauropsids (reptiles and birds), and hypothalamic-pituitary-interrenal (HPI), in amphibians and teleosts (**Figure 1**). This neuroendocrine axis is responsible for the stress response in all vertebrates (1). The neurohormone, corticotropin releasing hormone (CRH), controls the production and release of corticotropin (also named adrenocorticotropic hormone, ACTH), at the pituitary level. ACTH then stimulates the production and release of GC from the adrenal gland in amniotes (mammals and sauropsids) or the interrenal cells in amphibians and teleosts by binding on melanocortin receptor 2 (MC2R). GC act on diverse target tissues and also operate a negative feedback on the brain (hypothalamic CRH) and the pituitary (ACTH) (1–3) *via* specific receptors, the glucocorticoid receptors (GR) (4, 5).

Glucocorticoids are part of corticosteroids together with mineralocorticoids. GC are steroid hormones derived from cholesterol, as sex steroids are. In mammals and sauropsids, GC are synthetized by the adrenal cortex cells of the adrenal gland, while in amphibians and teleosts, GC are synthetized by the interrenal gland, a tissue embedded inside the anterior part of the kidney (head kidney) and homologous to the adrenal cortex of the mammalian adrenal gland (6). Cortisol is the primary GC in most mammals and teleosts, while corticosterone is the main in birds, reptiles, amphibians and many rodents (7). For easier reading, cortisol and corticosterone will be abbreviated, as glucocorticoids, by GC, throughout this review.

**Figure 2:**
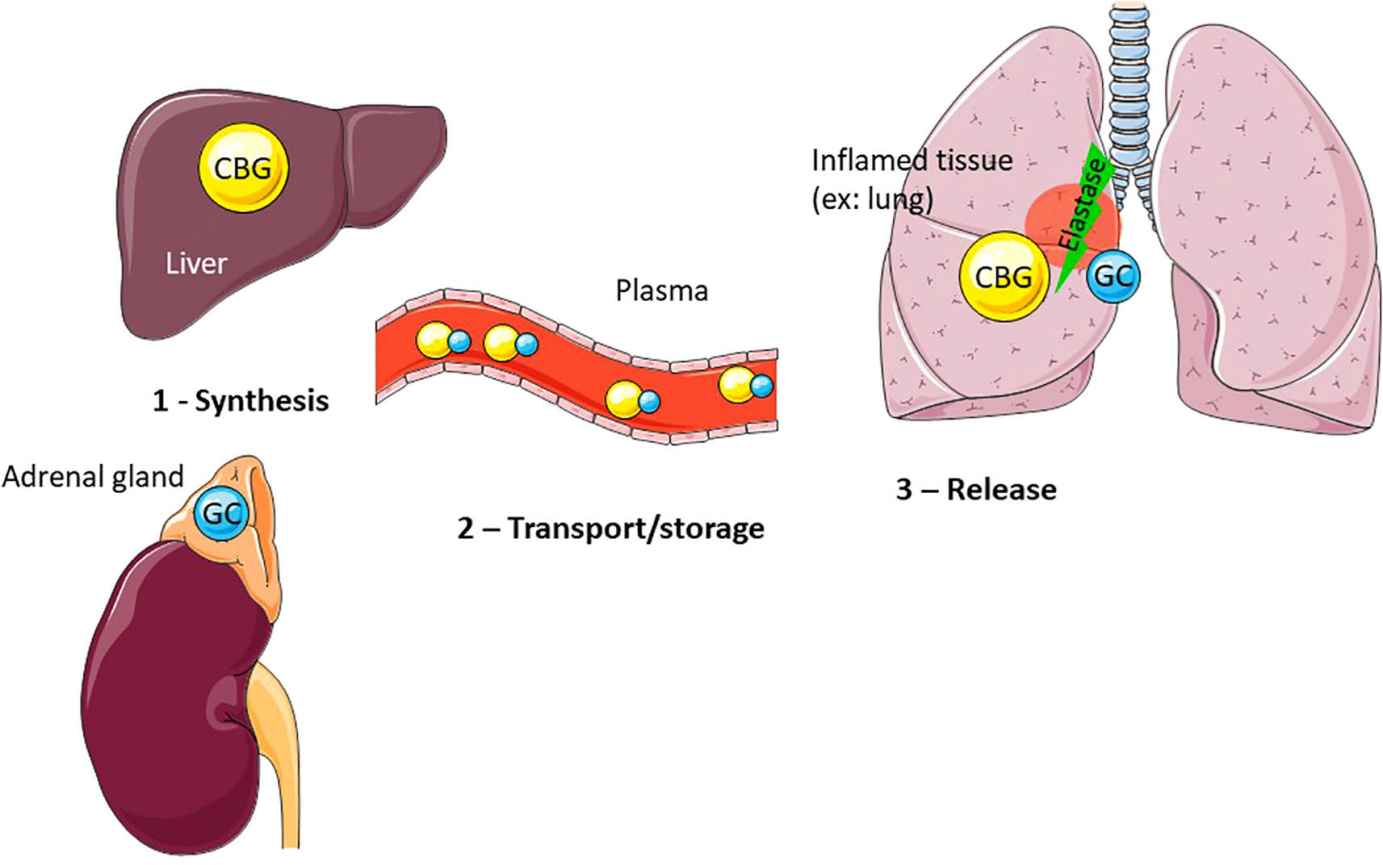
CBG mainly produced by the liver, and more specifically by the hepatocytes, is released in the plasma where it binds approximately 90% of GC. At the inflammatory site (example: lung), CBG is cleaved by activated neutrophil elastase, leading to a conformational change of CBG and resulting in the local release of bound GC.

As the axis regulating stress response, the HPA/HPI axis allows vertebrate adaptation to predictable and unpredictable changes in their environment (8–12). GC are thus key intermediaries between vertebrates and their environment. Increased baseline levels of GC are indicative, for example, of periods of increased energetic demand, such as during reproduction or during periods of low resource availability linked to life-history stages. An acute increase in GC is also observed in response to unpredictable environmental changes and reflects the ability of individual to maintain homeostasis. As in most vertebrates, plasma GC are bound to a binding protein named the corticosteroid binding globulin (CBG) (**Figures 1**, **2**), changes of this protein levels or binding affinity/capacity are predicted to have major impact on GC availability and actions.

Our review begins by a description of CBG discovery, characterization and measurement in vertebrates. A focus on the variations of CBG concentrations in various physiological conditions or under non-natural situations in vertebrates follows. The current knowledge on the different functions reported for CBG is then unfold. Our review ends with CBG alterations observed in human pathologies to evidence how this protein could have therapeutic uses.

## 2 CBG discovery, characterization, measurement and hormonal regulation in vertebrates

### 2.1 CBG discovery and characterization

Corticosteroid-binding globulin (CBG) was discovered in the human plasma in the 1950s by three different groups (13–15). The latter group called it transcortin by analogy to transferrin, the iron-binding protein (14). This CBG presents low capacity but high affinity for corticosteroids, which is in contrast to albumin, another plasma transporter protein, which has a high capacity but low affinity for these hormones (16, 17).

CBG is a protein consisting of 405 amino acids (aa) with a molecular weight of 45 kDa. Before its secretion into plasma, its first 22 aa, which represent the cellular export peptide signal, are excised. Thus, circulating CBG is composed of 383 aa with a theoretical weight of 42 kDa (18). However, since CBG is glycosylated, it is generally detected with a molecular weight between 60 and 70 kDa. CBG sequence contains 6 asparagines (Asn) corresponding to 6 N-glycosylation putative sites: Asn9, Asn74, Asn154, Asn238, Asn308 and Asn347 (the position of the Asn is indicated without the signal peptide). These glycosylations appear to have an important role in the function of the protein. Glycosylation is commonly considered as a message for intracellular trafficking and exportation. In the case of CBG, glycosylation may also be important for GC binding and delivery. For example, glycosylation at position 238, may ensure CBG binding to GC (19, 20) by allowing proper folding (19), while glycosylation at position 347 may be important in modulating GC delivery (21, 22). Thus, variations in protein glycosylation may cause variations in GC binding affinity and may protect CBG against proteolysis (23, 24).

Among vertebrates, mammalian CBG were the first to be characterized [human (18); rat (25); rabbit (26); sheep (27)]. The primary structure of human CBG defines it as a serine proteinase inhibitor (serpin) family member (18). Its gene (SERPINA6) is located in a cluster of related clade A SERPIN genes (28, 29) with synteny across other mammalian genomes (30). Phylogenetic studies showed that CBG is part of the SERPIN family (SERPINA6) (31–33). In birds, when looking for proteins able to bind steroid hormones in 23 avian species, Wingfield and colleagues discovered that no specific sex hormone-binding protein/globulin (SHBG) with high affinity and low capacity could be characterized in any of the species investigated (34). They also found that the high affinity binding protein for corticosterone binds progesterone with identical affinity, whereas testosterone and estradiol are bound with lower affinity (34). Birds were shown to lack SHBG gene and thus CBG is likely the substitute for SHBG in this vertebrate group (35). CBG characterization in white-throated sparrow (*Zonotrichia albicollis*) plasma showed that CBG binds corticosterone with over 6-fold higher affinity than testosterone (36). In amphibians, CBG binds GC and sex steroids with equivalently high affinity [*Ambystoma tigrinum* (37); *Bufo terrestris* (38)]. In reptiles, two steroid-binding globulins were identified in male tree lizard *Urosaurus ornatus*: one binds androgens and estradiol with high affinity and is similar to other vertebrate SHBG; the other binds androgens and C21 steroids (steroids containing 21 carbon atoms) including progesterone and corticosterone with higher specificity than estradiol and is named androgen-glucocorticoid-binding globulin (AGBG) (39). Genes for both plasma AGBG (named CBG in the article) and SHBG were identified in a study performing phylogenetic comparisons in anole lizard *Anolis carolinensis* and Chinese softshell turtle *Pelodiscus sinensis* (40). In teleost fish, little evidence for a plasma CBG-like protein exists (41), as most of the few data available reported a lack of specific plasma proteins able to bind cortisol (42–45). Only Caldwell and colleagues observed that mature female rainbow trout had greater cortisol bound to a CBG-like (48%) than mature males (16%) and immature fish (19.5%) (46), but this study relies on binding assay and not gene characterization, which is not sufficient for demonstrating the existence of CBG in teleosts. In contrast, SHBG genes have been characterized in this vertebrate group (47).

### 2.2 Measurement of CBG levels and binding capacity/affinity

Mainly synthetized by the liver (48), the CBG is released in the blood (**Figure 2**). CBG was measured, by means of gel-filtration method, in the plasma of 131 species representing all the vertebrate classes and was detected in every vertebrate species studied, even fish species (7, 49). In fact, at that time, it was the % of steroid (cortisol and corticosterone) bound which was assayed, and up to now no CBG has been characterized in teleost fish. Desantis and colleagues demonstrate a dichotomous pattern among vertebrates with respect to maximum corticosteroid binding capacity (MCBC) and total CBG levels (50): a dominant branch, where high levels of CBG bind most of the GC, applies to the majority of vertebrates; a secondary branch, comprising lamprey, flying squirrel or New World monkey, in which low levels of CBG bind almost none of the GC, applies to a very small subset. For the latter, the critical unknown is how these species mitigate the impact of the high free GC levels and how such a dramatic trait shift could evolve (50). Other comparative studies, this time among birds and mammals, also show a great variation in the binding capacity and affinity of CBG within and among the avian and marine mammalian species studied (51, 52). Beyl and collaborators recently evidence the importance of assay temperature when measuring CBG, as colder temperatures maximize specific binding but likely underestimate GC affinity for CBG (53).

### 2.3 Hormonal regulation of CBG levels

Although the regulation of CBG by GC has been widely studied in the literature, no consensus on this subject has emerged. Adrenalectomy induced a CBG increase in rats *Rattus norvegicus* (7, 54), while having no effect in the other mammalian species tested (human, *Homo sapiens*; guinea pig, *Cavia porcellus*; rabbit, *Oryctolagus cuniculus*; dog, *Felis familairis*; cat, *Felis domesticus*) (7, 55). No change in plasma CBG levels was noticed after injection of dexamethasone, a synthetic GC, followed by ACTH administration, in snowshoe hare *Lepus americanus* and laboratory rabbit (56) or of corticosterone in rat (57). A rapid increase of plasma CBG levels is observed after ACTH injection in rat (58) and laboratory rabbit (56). In male rat, subcutaneous injection of dexamethasone was, however, able to decrease both plasma and hepatic mRNA levels of CBG (59). Using *in vitro* perfused liver slices from adrenalectomized male rats injected intraperitoneally *in vivo* with GC, Feldman and colleagues demonstrated that both prednisolone and dexamethasone also induced a decrease in CBG basal concentration and production rate (60). A decrease of CBG mRNA levels was also observed using mouse hepatoma cell line (BWTG3) treated by dexamethasone (61). In contrast, dexamethasone treatment had no effect on CBG mRNA levels and release by mouse Leydig tumor cell line (mLTC-1) (62). In human, both endogenous and exogenous glucocorticoids can modulate circulating levels of CBG: plasma levels of CBG are suppressed during prolonged exposure to GC, whether endogenous, as in Cushing’s syndrome, or exogenous, as after administration of synthetic GC (55, 63). A decrease of CBG (this time mRNA levels) was also observed *in vitro* after treatment by dexamethasone of hepatoma cell lines from human (HepG2) (61). However, another study, also carried out on HepG2, showed no effect of dexamethasone on hepatic CBG synthesis, regardless of the dose used (64). Jung and colleagues also reported that the administration of hydrocortisone to healthy subjects, either orally or intravenously, does not change plasma CBG levels (65). Interestingly, one study showed that dexamethasone increased CBG production during fetal life, while inhibiting it in adulthood (27). Studies on the effect of glucocorticoids on CBG expression seem therefore dependent on the model, age, dose, and type of glucocorticoid used, with synthetic ones being more effective in regulating CBG.

In mammals, castration leads to either increase (rat; mouse, *Mus musculus*; cow, *Bos taurus*; goat, *Caprya hircus*), or decrease (rabbit; horse, *Equus caballus*) and even no change (human; cat; pig, *Sus scrofa*; hamster, *Mesocricetus auratus*) (7). Estrogen treatment was reported to increase CBG concentration in men and women (49, 55), as well as in some other mammals [rats, mice, rabbits and guinea-pigs (49)]. However, this was not the case in dogs (66) and sheep (67). The *in vitro* basal concentration and production rate of CBG by liver slices was also increased in adrenalectomized male rats injected intramuscularly with estradiol (60). In the rat, sex difference was observed concerning CBG activity, as administration of estradiol (E2) did not change it in females but increased it in males (68). Recently, the gonadotropin, luteinizing hormone, was shown to differentially regulate CBG mRNA levels (decrease) and release (increase) by mLTC-1 (62).

A decrease of CBG levels is observed in human hyperthyroidism (55). In contrast, in male rat, the thyroid hormone T4 administrated subcutaneously increased serum CBG and hepatic CBG mRNA levels, but did not influence the rate of CBG gene transcription, suggesting that T4 increases CBG mRNA stability (59).

## 3 Situations of CBG variations

### 3.1 Natural physiological variations

#### 3.1.1 Stress

Stress is a physiological reaction that encompasses all of an organism’s responses to external pressure in order to ensure survival. Acute stress, for example, induces an increase in glucocorticoid levels and could also cause changes in CBG levels, as described below and summarized in **Table 1**.

**Table 1:** Effects of various stressors on CBG in vertebrates.

Species	Sex	Stressors	CBG changes	References
Mammals				
Richardson’s ground squirrel *Urocitellus richardsonii*	Males	Live trapping	Decrease in MCBC	(72)
Arctic ground squirrel *Urocitellus parryii*	Males and Females	Live trapping	No change in MCBC	(76)
	Breeding males	ACTH injection	Decrease in MCBC	(72)
Red squirrel *Tamiasciurus hudsonicus*	Breeding males	ACTH injection	Decrease in MCBC	(72)
Snowhoe hare *Lepus americanus*	Males	ACTH injection	Increase in MCBC	(74)
	Experiment 1992: both sexes	ACTH injection	Increase in MCBC	(56)
	Experiment 1996: males		No increase in plasma CBG levels	
New Zealand white rabbit *Oryctolagus cuniculus*	Males	ACTH injection	Increase in MCBC	(56)
			No increase in plasma CBG levels	
Sprague-Dawley rat	Males	ACTH injection	Decrease in plasma CBG levels	(58)
	Males	Inescapable tail shock	Decrease in plasma CBG levels	(57, 78)
	Males	Immobilization	Decrease in serum CBG levels	(79)
	Males	Restraint	No change	(79)
		Food and water deprivation	No change	
Wistar rat	Females	Ether anesthesia	Decrease in serum CBG levels	(82)
		-swimming -swimming after fasting -fasting -fasting after cold exposure		
		-ice bathing -cold exposure	No change	
		-water deprivation	Increase in serum CBG levels	
	Males	Fasting	Decrease in serum CBG levels	(82)
		Fasting+cold exposure		
		Ether stress; ice-bathing; swimming; water deprivation	No change	
	Males	Physical restraint	Increase in plasma CBG levels	(80)
		Forced swimming	Increase in plasma CBG levels	
	Males	Involuntary swimming	Decrease in CBG mRNA levels	(61)
		Restraint		
		Running in wheels	No change in CBG mRNA levels	
PVG/C Lister Hooded male x WAG/C Wistar Albino female rat	Females	Fasting	Decrease in plasma CBG levels	(83)
	Males		No change	
Long-Evans rat	Males (and females)	Chronic social stress (15 days)	Decrease in plasma CBG levels	(84)
Little brown bat *Myotis lucifugus*	Females	Capture	No change	(75)
		Handling		
Birds				
Japanese quail *Coturnix japonica*	Males	Acute handling	No change in CBG binding capacity	(70)
European starling *Sturnus vulgaris*	Females	Acute handling	No change	(70)
	Non-breeding males and females	Chronic psychological stress (18 days): loud radio, cage trapping, cage rolling, human voice, bag restraint	No change in CBG capacity	(77)
White crowned sparrow *Zonotrichia leucophrys*	Males	Acute handling	No change (adults-nestlings)	(70)
	Males	Experimental fasting	Decrease in CBG binding capacity	(120)
House sparrow *Passer domesticus*	Males	Acute handling	No change	(70)
Red crossbill *Loxia curvirostra*	Males and females	Acute handling	Decrease	(70)
American kestrel *Falco sparverius*	Juveniles	Acute handling	Decrease	(70)
Laysan albatross *Phoebastria immutabilis*	Incubating females	Acute handling	Decrease	(70)
Zebra finch *Taeniopygia guttata*	Males	Acute handling	Decrease	(70)
Common stern *Sterna Hirundo*	Incubating males and females	Acute handling	Decrease	(70)
Eurasian tree sparrow *Passer montanus*	Males	Capture	Increase in plasma CBG capacity during second nestling stage	(73)
		Handling		
		Restraint		
	Females	Capture	Increase in plasma CBG capacity during second nestling stage	(73)
		Handling	Increase during egg-laying stage	
		Restraint	Decreased during building stage	
Barn owl *Tyto alba*	Nestling males and females	Experimental fasting	Increase in CBG capacity	(129)

##### 3.1.1.1 Free-living vertebrates

Free-living vertebrates need to have an appropriate stress response to be able to adapt to predictable and unpredictable changes in their environment. The necessity of measuring CBG, in addition to total GC, in natural populations of vertebrates to properly assess the impact of stress in wild populations has been underlined by Breuner, Delehanty and Boonstra in their review of 2013 (69). In a comparative study among birds using nine species from five orders and nine families, CBG capacity significantly declines within 30–60 min of acute stress (capture and handling) in five of the species investigated, leading to elevated free corticosterone levels in plasma and likely more corticosterone to reach tissues (70). Thus, the corticosterone may be available to play its role for the increased metabolic needs during stress. A decline of MCBC is also reported after acute stress in mammals. In Richardson’s ground squirrel *Urocitellus richardsonii* (previously named *Spermophilus richardsonii*) a 21% drop in MCBC is reported 4h after live trapping (71). Hormonal challenge with ACTH can also induces a decline in CBG levels in squirrels [red squirrel *Tamiasciurus hudsonicus* and arctic ground squirrel *Urocitellus parryii*, previously named *Spermophilus parryii* (72)].

CBG levels may also increase in response to a stressor, thereby decreasing the amount of circulating free GC. For example, in the multi-brooded bird, the Eurasian tree sparrow *Passer montanus*, CBG capacities can vary after acute stress (capture - handling - restraint) in males as in females, differently depending of the life history stages: an increase is observed during the second nestling stage in males, and during the second egg-laying and the second nestling stages in females, while a decrease is noted during the nest building stage in females (73). A rapid (30 min) increase of plasma CBG levels is observed in snowshoe hare *Lepus americanus* after ACTH injection, but not dexamethasone injection (56, 74).

MCBC does not show change after acute stress by capture and handling in little brown bat *Myotis lucifugus* (75) and by live trapping in a species of squirrels the arctic ground squirrel, no change is reported (76) (Boonstra et al., 2001). This lack of detectable changes in CBG was also reported in a bird, the European starling *Sturnus vulgaris*, after psychological stress (loud radio, cage tapping, cage rolling, human voice, and bag restraint) (77).

##### 3.1.1.2 Laboratory animals

Various studies in rodents have examined the effect of stress on CBG production and the release of corticosterone, the major GC in these models (**Table 1**). In rats, a decrease in plasma CBG levels is observed after inescapable tail shock (57, 78) and immobilization (79), but not after milder stressors such as restraint and food/water deprivation (79). Qian and colleagues show that in cases of intense stress (here, physical restraint or forced swimming), CBG is released into the plasma from rat livers within the first 15 minutes following the stressful event (80). The researchers observed a time delay between the increase in total corticosterone and that of free corticosterone. The release of CBG therefore introduces a delayed response and thus builds up a glucocorticoid reserve. Longer-term study (10 days) indicate that hepatic CBG mRNA levels are decreased after physiological stressors such as involuntary swim exercise or restraint, but not after run in wheels (61). However, the stressful event must be long and intense, and the decrease in plasma CBG will only be observed 6 h after the induced stress and up to 72 h after (57, 79, 81). Various types of stressful events are also capable of inducing this decrease. Tinnikov exposed rats to so-called classic stresses (forced swimming or ether anesthesia) and metabolic stresses (fasting or ice baths) and observed a decrease in plasma CBG regardless of the type of stress, in females (82). Interestingly, in males, no change in CBG levels is reported after fasting (83). In males, a large reduction in plasma CBG levels is observed during social stress in subordinate (around 70%) and dominant (around 40%) rats compared to controls rats (84).

#### 3.1.2 Strains in rodents

Differences also exist between rodent strains. A genetic study revealed that C57BL/6 mice were more sensitive to a pro-inflammatory challenge with TNF-α than DBA/2 mice, and that this trait was linked to the *Serpina6* locus (85). It has also recently been shown that Sprague Dawley Harlan rats have lower plasma CBG levels compared to Charles River rats (86). Harlan rats are also more sensitive to a pro-inflammatory challenge in an induced arthritis model or after treatment with LPS (86, 87). A lower level of corticosterone in Harlan rats could thus be the basis for a greater susceptibility to inflammation. All of these studies tend to show the key role of CBG in controlling the inflammatory response. Another study in rats also revealed the presence of polymorphisms in the *Serpina6* gene that influence the affinity of CBG for corticosterone. Smith and Hammond revealed that BioBreeding rats, derived from Wistar rats, have a 50% lower affinity for corticosterone compared to Wistar rats (88). Comparison of CBG cDNA showed them that there is a polymorphism in BioBreeding rats causing the substitution of methionine 276 for isoleucine, which is the cause of the reduced affinity. The same types of genetic variants have been found in humans, where the consequences for the protein are diverse.

#### 3.1.3 Life-history stages

It is well known that GC rise during development in many species across different taxa in order to assist with transitions between life history stages (5, 89).

##### 3.1.3.1 Pregnancy in placental mammals and breeding season in other vertebrates

Elevations in GC are seen during gestation and breeding season, as well as prior to parturition or hatching in most mammals, birds, lizards, and large fish species (5, 89). Potential roles of GC in late gestation have been suggested: -role in the onset of parturition; -role for the increased energetic needs by the mother; -provide an indication of the conditions in the external environment that the fetus will encounter; -role in fetal development/fetal organ maturation (90). Changes in CBG can contribute to the variation of GC observed during this life-history period that is crucial for species survival (**Table 2**).

**Table 2:** Variations of CBG during life-history stages.

CBG variation	At which stage/period?	Sex	Pregnancy - Breeding season	Other stages/periods studied	References
Increase			With increase in total GC		
	pregnant	women	Human *Homo sapiens*	non pregnant	(14, 92–94)
	pregnant	females	Mouse *Mus musculus*	non pregnant	(95)
	pregnant	females	Guinea pig *Cavia porcellus*	non pregnant	(95)
	from day 23 to 27 of gestation	females	Domestic rabbit *Oryctolagus cuniculus*	from day 11 of gestation to 3 days after delivery	(26)
	pregnant and lactating	females	Meadow vole *Microtus pennsylvanicus*	non perforate; perforate; pregnant; lactating	(96)
	breeding season	both	Gambel’s white-crowned sparrow *Zonotrichia leucophrys*	winter season; migration season	(109)
	breeding	both	Lapland longspur *Calcarius lapponicus*	molt	(108)
	laying	females	European starling *Sturnus vulgaris*	incubating; chick rearing	(106)
	prior to egg-laying	both	Tufted puffin *Fratercula cirrhata*	late incubation; late chick-rearing	(111)
	between 60 and 120 days of pregnancy	females	Baboon *Papio papio*	non-pregnant; at term	(98)
	between 60 and 140 days of pregnancy	females	Rhesus macaque *Macaca mulatta*	between 140 and 167 days of pregnancy	(97)
	visibly pregnant and lactating	females	Arctic ground squirrel *Urocitellus parryii*	not visibly pregnant	(99)
			Total GC not measured		
	beginning of breeding season	males	Dark eyed junco *Junco hyemalis*	end of breeding season	(105)
	during nest building, first egg-laying and first nestling stages	males	Eurasian tree sparrow *Passer montanus*	late wintering; first nestling; second egg-laying; second nestling; pre basic molt	(73)
	during nest building stage	females	Eurasian tree sparrow *Passer montanus*	late wintering; first egg-laying; first nestling; second egg-laying; second nestling; pre basic molt	(73)
Decrease			With increase in total GC		
	pre-nest building	both	Pied flycatcher *Ficedula hypoleuca*	nestling	(107)
			With no change in total GC		
	from 20 days of pregnancy	females	Laboratory rats	throughout pregnancy	(100)
	pregnant	females	Horse (mare) *Equus ferus*	non pregnant	(101)
No change	pregnant	females	Cow *Bos taurus*	non pregnant	(102)
	pregnant	females	Domestic pig *Sus scrofa*	non pregnant	(102)
	early gestation	females	Richardson’s ground squirrel *Urocitellus richardsonii*	late gestation	(103)
	incubation	unknown	Black legged kittiwake *Rissa tridactyla*	early chick rearing; late chick rearing	(110)
CBG variation	At which stage/period?	Sex	Life-history stages implying fasting	Other stages/periods studied	References
Increase	fasting	males	Ice-free period – polar bear *Ursus maritimus*	feeding	(118)
Decrease	hibernation state	subadult females and males	Hibernation – brown bear *Ursus arctos*	active state	(119)
	unfed	unknown	Fledging – Laysan albatross *Phoebastria ammutabilis*	fed	(122)

###### 3.1.3.1.1 Pregnancy in placental mammals

In their discovery paper of 1959, Slaunwhite and Sandberg already showed that the plasma concentration of CBG considerably increased during the third trimester of pregnancy in human (14). It was then shown that CBG capacity is increased by 3 times in pregnant women, as plasma E2 levels increase (91–93).

In their review, Edwards and Boonstra compiled all the blood-based studies reporting total GC from 33 mammalian species during pregnancy (90). In these 33 species, CBG was measured in only 12 cases. An increase in total GC production associated with an increase in CBG was observed in humans (92–94), guinea pigs *Cavia porcellus* (95), mice *Mus musculus* (95), meadow voles *Microtus pennsylvanicus* (96) and domestic rabbits *Octolagus cuniculus* (26, 95). No change in total GC production but an increase in CBG was reported in macaques *Macaca mulatta* (97), baboons *Papio hamadryas* (98) and arctic ground squirrels (99). In addition, no change in total GC with a decrease in CBG was evidenced in laboratory rats *Rattus norvegicus* (100) and horses (mares) *Equus ferus* (101). In two mammalian species, belonging to Artiodactyls, both maternal total GC and CBG do not change over pregnancy [cow *Bos taurus* and domestic pig *Sus scrofa* (102)]. In the Richardson’s ground squirrel, their levels also do not differ between early and late pregnancy (103). The authors argue that the absence of changes in maternal total GC and CBG during pregnancy is likely due to the maturity of the fetal adrenals in this order, leading the fetus to produce the majority of its own GC in late gestation (90). Arctic ground squirrels present an interesting case of reduced free GC during pregnancy (99). Females of this species have to cope with pregnancy in freezing temperatures and limited food availability after 9 month-hibernation. To be able to succeed, they encounter CBG buffering of high maternal stress as shown by Edwards and Boonstra (99). Indeed, females at three different life stages (not visibly pregnant, visibly pregnant and lactating) show similar total cortisol levels, but 4-fold increased CBG levels when visibly pregnant and lactating, resulting in a decline of free cortisol from 51% in not-visibly pregnant females to 5% in visibly pregnant and 10% in lactating (99). The authors postulate for “a seasonal adaptation relating either to the pronounced physiological changes the female must undergo after emerging from hibernation and immediately getting pregnant, or to the mobilization of body reserves for energy to permit pregnancy, or both”. High CBG levels may thus protect the developing offspring from the negative effects of GC overexposure.

###### 3.1.3.1.2 Breeding season in other vertebrates

Seal and Doe first reported that egg-laying amphibians, reptiles and birds do not present the increase in plasma CBG concentration observed in some placental mammals (7). Thus, free GC concentrations in free-living reptiles, amphibians, and birds are commonly elevated during the breeding season (104). These high levels of GC would have energetic and behavioral effects, as well as a role in preparing the animal for subsequent stressors (104).

In many short-lived birds, CBG may be modulated in relation to reproductive stage [dark eyed junco *Junco hyemalis* (105); European starling *Sturnus vulgaris* (106); pied flycatcher *Ficedula hypoleuca* (107); Lapland longspur *Calcarius lapponicus* (108); Gambel’s white-crowned sparrow *Zonotrichia leucophrys* (109)]. In contrast, in long-lived seabirds, such as the black-legged kittiwake *Rissa tridactyla*, there is a lack of consistent reproductive patterns in CBG levels (110). This difference likely resides in the fact that short-lived species have only few opportunities to reproduce and thus must invest heavily in each reproductive attempt.

Williams and collaborators compared the dynamics of CBG, total GC, and free GC in breeding tufted puffins (*Fratercula cirrhata*) from two different colonies with different rates of nestling growth and survival (high versus low productivity) during 2 years (111). They report that at the high productivity colony, levels of CBG, total baseline GC, free baseline GC, and total maximum GC were all higher prior to egg-laying than during late incubation and late chick-rearing. Levels of CBG were positively correlated with body condition index (BCI) and free baseline GC was negatively correlated with BCI. Total baseline levels of GC during chick-rearing were two to four times higher at the colony with low rates of nestling growth and survival. Tree sparrows show also variations in baseline CBG according to life stages: male sparrows have higher CBG capacities during the nest building, the first egg-laying and the first nestling stages, while females present this increase only during the nest building stage (73). Thus, birds can have adaptative strategies *via* seasonal fluctuations of baseline CBG in order to optimize their physiological and behavioral states to the life history cycle.

In birds as both sex steroids and GC can bind CBG with high affinity, the physiology (actions and metabolism) of testosterone (T) could be affected by both CBG and GC. During breeding season, the white-throated sparrow *Zonotrichia albicolis* exhibits unique behavioral and discrete plumage polymorphisms that are manifested in both sexes. White-striped (WS) morphs respond more aggressively to simulated territorial intrusion and tan-striped (TS) morphs provision nestlings at a higher rate (36). A difference in total T between male morphs (112) has been shown, but no sex nor morph differences in CBG has been observed (36).

In tree lizards, alternative male reproductive tactics correlated with throat-fan coloration exist: orange-blue males are aggressive and territorial, while orange males are non-territorial. Jennings and colleagues reported that AGBG capacity is significantly greater in territorial than non-territorial males, which could lead to higher levels of free corticosterone in non-territorial males than in territorial males, especially during stress-induced increases in corticosterone (39). As, in contrast, the capacity of SHBG does not differ between the two types of males, this may explain why testosterone levels of non-territorial males are more sensitive to negative feedback by corticosterone (39).

##### 3.1.3.2 Life transitions implying fasting

###### 3.1.3.2.1 Natural extended fasting

Most vertebrate species with regular seasonal fasting have lower serum total corticosteroid levels during fasting, which may lead to suppressed catabolic processes and behaviors necessary for survival. Some examples are king penguins (113, 114), migratory birds (115), elephant (116) and fur seals (117).

####### 3.1.3.2.1.1 During ice-free period and hibernation in bears

Polar bears *Ursus maritimus* also experience natural extended fasting during the ice-free season when they are forced ashore, but no change in total serum cortisol is observed in fasting compared to feeding animals (118). However, an elevated serum CBG expression (**Table 2**) is reported in fasting polar bears, which reduces free cortisol levels and contribute to fasting adaptation to decreased target tissue response to cortisol exposure, like for example downregulation of protein catabolism and amino acid mobilization (118).

During hibernation, brown bears *Ursus arctos* have higher levels of metabolically active GC and low CBG (**Table 2**) and Frфbert and colleagues suggest that high glucocorticoid activity likely promote lipolysis and gluconeogenesis while limiting tissue glucose uptake to maintain a continuous glucose supply to the brain in order to support the hibernation state (119).

####### 3.1.3.2.1.2 Before fledging in Laysan albatross

In birds, GC have also been shown to be important mediator of the transition to independence, such as fledging [white stork (120); pied flycatcher (121)].

Laysan albatross (*Phoebastria ammutabilis*) chicks increase their body mass to 150% of adult one during post-hatching period, before fasting when they approach fledging. They thus lose weight as energy is put into feather growth and wing development. Plasma GC levels increase during this fasting period while CBG levels decline (**Table 2**), which amplify free GC before fledging, and chicks which present the higher free GC levels fledge sooner (122). If chicks are fed artificially during the month before fledging, they stay at the colony longer as they show slower decrease of body mass, slower CBG decline and slower free GC increase (122). The authors conclude that free GC acts as a signal of energetic or nutritional state to adjust the time of fledging.

###### 3.1.3.2.2 Forced fasting due to natural environmental perturbations

Inclement weather can cause free-living animals to experience decreased food availability, extreme fluctuations of temperature and damaged habitats. Seasonally breeding birds are particularly sensitive to such unpredictable environmental perturbations and they can stop breeding. In such deleterious conditions, an increase of GC plasma levels in the field has been reported, associated with an increase of locomotor activity [Lapland longspur (123); common diving petrel *Pelecanoides urinatrix* (124); song sparrow: *Melospiza melodia* (125); white-crowned sparrow (126, 127)]. When wild-caught captive male Gambel’s white crowned sparrows, housed in photoperiodic conditions mimicking breeding season daylength, are submitted to acute, short-term fasting such as they may encounter during the initial stages of a severe storm at the onset of the breeding season, their CBG capacity is reduced leading to elevated free GC levels and their locomotor activity increased (127). The authors suggest that under low food conditions, GC secretion may be enhanced in order to increase the foraging and food searching behaviors of the birds. Nevertheless, if these low food conditions persist, CBG binding capacity drops and thus free GC peaks to enhance GC metabolic actions in order to ensure survival. In nestling barn owls *Tyto alba* raised under poor environmental (feeding) conditions, high total corticosterone and high CBG capacity and thus low free GC levels [which were calculated according to the equation from Barsano and Bauman (128)] are reported compared to nestlings experimentally fed *ad libitum* (129). When nestlings fed *ad libitum* are implanted a corticosterone-releasing pellet, total corticosterone, CBG capacity and free GC levels do not change compared to nestlings fed ad libitum and implanted with a placebo pellet, while they increase in nestlings receiving GC implant in low feeding conditions (129). The authors suggest that “the role of CBG varies with environmental conditions. Under more risky conditions, CBG may act as a buffer to avoid high free corticosterone levels as a result of repeated environmental perturbations. Corticosterone administration (by implant here) induces an increase in CBG capacity only in poor environmental conditions when the increase in total GC is quite high” (129). As these last two studies use experimental fasting, they are equivalent of a stressor and they appear in **Table 1**.

The role of CBG as a buffer may also be seen in other environmental challenges that do not imply fasting. It is the case during urbanization of birds [adult male songbirds: house sparrow *Passer domesticus*, Northern mockingbird *Mimus polyglottus*, curve-billed trasher *Toxostoma curvirostre*, Albert’s towhee *Pipilo aberti*, Canyon towhee *Pipilo fuscus* (130)] or in birds living in harsh environment [house sparrows living in New Mexico, a semi-arid area where they are obligate human commensals (131)].

In conclusion, natural variations in CBG can be encountered in numerous physiological situations such as stress, strain or reproductive stage.

As mentioned by John Hunter (1728–1793) in Treatise on the Blood, Inflammation and Gunshot Wounds, “inflammation is itself not to be considered as a disease but as a salutary operation consequent either to some violence or to some disease”. Despite this accurate definition of inflammation and for more clarity, this part will be unfold in section 4.2.Pro-inflammatory diseases.

### 3.2 Non-natural CBG alterations

#### 3.2.1 Changes of CBG due to environmental pollution

During chronic pollution by coal combustion waste (housing in mesocosm containing ash sediment) of southern toads *Bufo terrestris*, CBG increased from two to five weeks of experiment as in control groups (housed in mesocosm covered with control sand sediment), while total GC was only significantly elevated at four weeks (38). The increase in CBG did not parallel the increase in total GC; as a result, free GC levels were not buffered by CBG, but showed a peak at four weeks similar to total GC, indicating that in this species, CBG may not provide a protective mechanism during long-term pollution exposure (38).

At the No-Observed-Effect-Level (NOEL), approved for Australian fresh water residues and by the World Health Organization (WHO), both atrazin and fenitrothion compete with GC for CBG binding sites in cane toad and rat plasma (132). These agro-chemicals are thus competitively inhibiting the binding of GC to CBG, affecting the total/free ratio of GC and consequently disrupting the normal stress response (132).

#### 3.2.2 Human CBG-deficient patients and mouse model of CBG deficiency

Previous reviews have already described in detail genotype-phenotype associations for CBG in human and animal models (133–135).

##### 3.2.2.1 Human CBG-deficient patients

Very rare cases of SERPINA6 gene mutations have been found in patients with low total plasma cortisol levels, associated with various clinical manifestations including fatigue and chronic pain. To date, the literature reports the existence of 9 mutations that have consequences on either the affinity (and/or binding capacity) of the protein for its ligands or its plasma levels (136, 137).

The first CBG mutation was identified in 1982 in 3 individuals from 3 different families and was named transcortin Leuven (138). Although plasma CBG levels are normal, the CBG produced has a 3-fold lower affinity for cortisol than normal CBG. This loss of affinity results from a mutation that leads to the substitution of a leucine for a histidine at residue 93 (139, 140). This mutation was detected in patients with acute inflammatory diseases (140). The Lyon mutation, another mutation characterized by a loss of affinity of CBG for cortisol, has been more studied. Emptoz-Bonneton and colleagues report the case of a woman affected by this mutation who presented with chronic asthenia and hypotension, and who developed depression (141). Plasma CBG levels are lowered, and the affinity of this CBG for cortisol is reduced by 4 times compared to normal values. The authors also noted a decrease in the plasma level of total cortisol, while its free fraction is increased. These same results were found in a woman also possessing this mutation and who presented with asthenia and chronic drowsiness (142). Severe muscle fatigue was noted in a patient heterozygous for a variant of CBG Lyon mutation (143). Since then, many other mutations causing a loss of affinity and the same symptoms have been discovered in various individuals. A novel homozygous c.776g>t transversion in exon 3 of the CBG (*SERPINA6*) gene, resulting in a p.Gly237Val substitution, that is predicted to influence the positioning of two β-sheets that constitute part of the CBG steroid-binding site, was discovered in a 26-yr-old female with hypotension and fatigue and named CBG G237V (144). In a greek woman, heterozygous for single-nucleotide polymorphisms encoding the CBG Lyon (D367N) and CBG A224S variants, Hill and colleagues found a novel heterozygous c.1282G>C transversion in exon 5 of *SERPINA6*, resulting in a p.Trp393Ser (W371S) substitution, and named CBG Athens (145). Substitution of a Leucine by a Histidine at residue 93 also results in reduced affinity for cortisol; this mutation was named CBG A224S (140, 146).

Other types of mutation are capable of altering the amount of CBG in plasma. The Null/Adelaide mutation causes a total absence of the protein in the plasma of patients homozygous for this mutation (147). Patients suffer from hypotension and fatigue and have a decreased plasma level of total and free cortisol. Other mutations affecting the detected plasma CBG level have been identified, including the Santiago mutation (148). Patients heterozygous for this mutation have a plasma CBG concentration reduced by 50%. Patients also complain of chronic pain and fatigue, particularly after exercise. It should be noted that exogenous administration of GCs does not alleviate these symptoms, thus highlighting the important role of CBG targeting. In a village in Southern Italy, the Null/Adelaide and Lyon mutations were highly prevalent (149). A 39-member Italian-Australian family, with signs of fatigue and relative hypotension, also presents both Null/Adelaide and Lyon mutations (147). More recently, a clinically novel SERPINA6 mutation, CBG Montevideo, results in 50% reduced plasma CBG levels and was associated with low serum total cortisol, hypoglycemia, chronic fatigue and hypotension (137).

In Chinese population, two nonsynonymous single nucleotide polymorphisms were identified within *SERPINA6* exon 2 encoding CBG A51V and CBG E102G variants, as well as two nonsynonymous SNP encoding CBGs R64Q and R64W; CBG A51V bound steroid normally, but its production/secretion was severely impaired; CBG E102G was produced normally, but its cortisol-binding capacity was abnormally low, whereas CBG R64Q and R64W were produced and bound cortisol normally (150).

Unbiased genetic analyses were performed to identify the genetic factors influencing GC levels. A first study, conducted in pig models, proposed CBG gene as an interesting positional and functional candidate to explain the influence of the quantitative trait locus on plasma cortisol levels (151). Indeed, they found a highly significant gene effect for post-stress cortisol level and a significant effect for basal cortisol level at the end of the q arm of chromosome 7, region in which CBG gene is mapped in pig. Moisan’s group later presented experimental evidence that CBG gene was the major genetic factor explaining the variations in cortisol levels (152). This result was also observed in a rat model but only for stress-induced cortisol levels (153). Then, it was again detected in a human cohort, conducted on 12,597 subjects, showing that certain allelic variants of the *SERPINA6* gene are associated with lower plasma cortisol levels in the morning (154). Some of these polymorphisms have also been associated with alterations in plasma CBG levels. All of these results were replicated in a cohort of 1,077 adolescents (155). CBG therefore plays a central role in the variability of plasma cortisol levels. CBG also appears to play a role in the distribution of fat mass. Studies have negatively correlated plasma CBG levels with body mass index, waist-to-hip ratio, and insulin resistance (156). An allele of the *SERPINA6* gene appears to be involved in the correlation with the waist/hip ratio in obese women (157). In men also, this same allelic variant (CBG allele 90) was found to be increased in patients with morbid obesity compared to the rest of the population (30% versus 18%, p = 0.02) (158). This polymorphism also influences body mass index and waist circumference. Patients with this allele also have a tendency to decrease plasma CBG levels. Altogether these studies pointed out the importance of genetic interindividual variability to explain GC level variability. One of the best examples is the sexual dimorphism observed in plasma CBG with higher levels in female compared to male [human (159, 160); rat (161); mouse (162–165)].

##### 3.2.2.2 Mouse model of CBG deficiency

The idea that CBG plays an important role in the inflammatory response is supported by various studies on animal models deficient for CBG. Variations in its levels and its affinity for its ligands, already observed in patients with a *Serpina6* gene mutation, have also provided insight into the functions of CBG. The first *Serpina6* knockout (KO) mouse model was developed in 2006 (166), followed by another one in 2010 (167). These mice were viable, fertile, and do not present any phenotypic abnormalities or detectable architectural differences in the liver, kidneys, lungs, thymus, and adrenal gland, suggesting that CBG is not necessary for survival and has no critical role in the development of these organs (166).

Concerning the free plasma corticosterone levels of these KO mice, at rest, they are slightly increased in the morning at the nadir of GC secretion (166, 167) and unchanged in the evening at the beginning of the active phase (167). These data argue that at rest CBG deficiency has no or very little impact. In contrast, after a stress, the free plasma corticosterone levels are reduced in CBG-deficient mice compared to wild-type animals [restraint stress (167); forced swim test (162)]. This decrease in free corticosterone in the absence of CBG allows for better performance in memory task under stressful conditions and a reduced emotional response in females (168, 169). CBG therefore influences brain functions that drive stress responses, and these studies in animal models provide a foundation for understanding the stress-related mood and behaviour in human with CBG mutations (135). CBG deficiency impairs contextual and recognition memory consolidation in male mice (170). It also triggers metabolic imbalance in the hippocampus likely to cause brain damage and long-term neurological pathologies (171).

Concerning inflammation, Petersen and colleagues demonstrate that CBG-deficient mice are more susceptible to septic shock: injection of *Salmonella enterica* LPS (lipopolysaccharide) is responsible for a decrease in the survival of these mice, compared to heterozygous mice. This was accompanied by an increase in cytokine levels in the plasma and lung, where the infiltration of monocytes is abnormally high (166). These results strongly support the importance of CBG in the control of inflammatory response to infectious challenge. Using an experimental model of acute pancreatitis showing expected progressive inflammation, Gulfo and colleagues report that the lack of CBG does not abolish the increase GC levels in response to inflammation (164). It is also shown that hepatocyte Kruppel-like factor 15 controls inflammatory responses via direct activation of *Serpina6* gene promoter (172).

The sexual dimorphism observed in plasma total GC levels (higher in females than in males) is abolished by CBG deficiency (164) likely due to a stronger reduction of the adrenal expression of the main enzymes involved in GC synthesis in females (165). In CBG-deficient male mice fed an hyperlipidic diet, lipid partitioning is driven from subcutaneous to visceral adipose tissue leading to obesity, without affecting food intake and body weight (173). A number of adrenal (174) and hepatic (175) genes is altered by the loss of CBG specifically in female adult rats, suggesting that CBG is involved in the sexual dimorphism observed in the development and function of rat adrenal gland and liver.

## 4 Different known roles of CBG

### 4.1 CBG regulates the bioavailability of its ligands

As one of the main functions of CBG is to sequester GC in plasma by high affinity binding, it has long been considered under the prism of the free hormone hypothesis. This hypothesis asserts that the only biologically active part of the hormone is that which is free, i.e. not bound to a protein in plasma (176). Thus, the concentration of a hormone in a tissue is determined solely by its free plasma concentration, rather than by the concentration bound to its transport protein. This hypothesis is one of the best explanations for the clinical manifestations seen in patients suffering from hormone deficiency or excess. According to this hypothesis, the main function of CBG is to regulate the bioavailability and metabolic clearance of GC. A clinical study showed that patients with high plasma CBG levels cleared injected radiolabeled cortisol more slowly than those with lower levels (177). CBG therefore increases the half-life of plasma GC. Three studies in 2011 gave strong experimental evidence for this ‘free hormone hypothesis’. The first one was carried out in patients with a CBG mutation rendering it unable to bind its ligands (178). These patients had increased metabolic clearance and a decreased cortisol half-life. The second one assayed free GC levels in rats during and after various stressors (80). Forced swim stress induced an elevation of total plasma GC within 30 min, while the elevation of free plasma and tissue GC level was observed 30 min later (80). The third one showed that salivary GC levels mimic free plasma GC levels and both correspond to about 10% of plasma total GC (179). Thus, total GC is not what is available to tissue, but free GC (not bound to CBG) is.

In their review in 2013, Breuner and colleagues refer to a ‘reservoir hormone hypothesis’, a complimentary to the ‘free hormone hypothesis’, when citing Malisch and Breuner data on steroid-binding protein and free steroids in birds from 2010 (69). These authors suggest that the GC remaining bound serves as a reservoir of GC in the blood to be used as needed (180). In her correspondence paper in Nature reviews, Marie-Pierre Moisan gives different arguments for considering CBG as a cortisol reservoir rather than a transporter (181). First, cortisol can circulate in CBG-deficient patients (134) or in vertebrate species lacking CBG (133, 182), and the presence of CBG is necessary to mount a normal stress response as also shown by studies in CBG-deficient mice (167, 168). Signs of hypocortisolism rather than hypercortisolism are reported in case of CBG deficiency (please refer to section 2.2.2). She also mentioned that the mineralocorticoid, aldosterone, is equally hydrophobic as cortisol and does not possess a specific binding protein, as at that time only binding studies have reported a potential plasma binding proteins for this corticosteroid [human (183, 184)], which is still true twenty years after. For Marie-Pierre Moisan, it is albumin which ensures the transport of GC, as GC are mainly bound to albumin in CBG-deficient patients (141) or mice (167), and albumin is present in the blood of all vertebrates. Thus, CBG would have appeared during evolution to be the retention in blood of a circulating GC reserve readily available in case of an emergency (reservoir).

Various parameters can influence hormone distribution at tissue level. For example, the location of the target cell, endothelial permeability, the composition of the extracellular matrix and the juxtaposition of different cell types within the same tissue can influence a cell’s accessibility to a given hormone (185). The level of free cortisol is therefore not the only parameter determining its tissue concentration. CBG could play an active role in determining this concentration, notably through its addressing function.

### 4.2 CBG transports and addresses its ligands

In human, CBG circulates in low quantities (30 to 52 pg/mL) in plasma compared to albumin (40 g/L). However, its high affinity for GC means that it plays an important role in determining plasma GC concentration. CBG binds 80% to 90% of circulating cortisol, while 7% to 15% is bound to albumin and less than 5% is free (186).

CBG binds cortisol at a surface pocket located between the β B leaflet and the helix A and H. Like other serpins, CBG establishes a covalent bond with its ligand, which stabilizes CBG in its S conformation and exposes its reactive center loop (RCL). When cleaved by neutrophil elastase (**Figure 2**) between Val344 and Thr345, a conformational change in CBG occurs. The cleaved segment of RCL is then inserted into the β A leaflet, the protein is stabilized in its R conformation and loses its affinity for cortisol (187). With a 10-fold decrease in affinity, CBG releases cortisol. It has been shown that *Pseudomonas aeruginosa* elastase (LasB) can also cleave CBG at a site a few amino acids distant from the neutrophil elastase site (188). LasB is released by the bacterium at the sites it infects, while neutrophils release elastase at inflammatory zones. It is therefore considered that, in addition to its role as a transporter, CBG is able to specifically address glucocorticoids to the site of inflammation and infection. Thus, CBG could represent an interesting target to control exclusively GC effects on inflammation.

The binding of CBG to cortisol is also temperature-dependent. Several studies have shown a decrease in affinity between these two molecules as temperature rises from 37 °C to 42 °C (189, 190). This is consistent with the addressing role of CBG. Patients with inflammation or fever will have an increase in body temperature, allowing them to release more cortisol and resolve the inflammation as best as they can.

It is worth noting that chymotrypsin, a protease secreted by the pancreas, has been shown to be able to cleave the RCL of CBG (191). The discovery of this cleavage site is to this day still misunderstood, and its physiological significance remains to be elucidated.

### 4.3 Extrahepatic CBG controls the accessibility of ligands to their receptors

In addition to the liver, CBG is expressed (at lower levels) by various other organs and tissues [for reviews in mammals (192, 193)]. In mammals, its protein or transcripts have thus been detected in the kidney [rat (194); rhesus monkey (18); mouse (195)], the lung [rabbit (26); mouse (164)], the heart [human (196)], the spleen [rabbit (26)], the white adipose tissue [rat (197)], the ovary [rhesus monkey (18); rabbit (26)], the female genital tract [rat (194) and human (198) uterus; human Fallopian tubes (199)], the placenta [human (200)] and the testis [rhesus monkey (18) mouse (201)]. CBG is also detected in the adrenal [mouse (165) and thyroid [rat (194)] glands, as well as the central nervous system [rat (202–204); mouse (205)] and the pituitary [guinea pig (206); rat (194)]. In a bird, the zebra finch *Taeniopygia guttata*, the CBG mRNAs were quantified in spleen, lung, kidney and gonads of males and females (while being undetectable in skeletal muscle), but their levels were more than 300-fold lower than those in liver (40).

Few studies have examined the role of these extrahepatic CBGs. It is commonly assumed that these CBGs do not contribute to plasma CBG levels, as they are intracellular. Despite their low level of expression, these CBGs could be finely regulated at the cellular level and play an important local role. In human lung, the levels of CBG transcripts seem to be differentially expressed among the airways and regulated in lung disease situation [cystic fibrosis (207)]. Concerning local CBG function, it is hypothesized that GC released from plasma would enter the cell and, at this level, bind intracellular CBG. Blocked in this way, the GC would no longer be able to bind its receptor, the main mediator of its intracellular actions, and would no longer be able to act.

This hypothetical role of extrahepatic CBG is supported by the better-known regulation of other hormones by their binding proteins. This is the case for IGF (insulin-like growth factor), which is transported in biological fluids by proteins known as IGFBPs (IGF binding proteins). They are capable of regulating the bioavailability of IGF, but also of modulating its activity by binding it locally to target tissues (208). CBG may enable equally fine regulation of GC. Depending on the tissues expressing these CBGs, limiting the intracellular actions of GC may have wider consequences. In the brain, CBG is expressed by numerous cell types, including astrocytes (209). GC are capable of inducing remodeling of these cells and eventually their death. Locally produced CBG could therefore act as a buffer to protect the cell from the deleterious effects of these molecules. CBG also appears to have a role in behavior and memory, particularly in response to stress (168). The role of CBG at the cerebral level in this emotional response remains to be determined, but it could modulate and control this stress response. In the lung, CBG could influence fetal maturation of this organ, particularly at the alveolar level. The differentiation of alveolar epithelial cells and the control of cell proliferation are indeed dependent on the action of GC (210). Pulmonary CBG could therefore also play a role in lung development and maturation. The putative roles of these local productions, however, remain to be confirmed in order to fully understand their biological relevance.

### 4.4 CBG triggers intracellular signaling cascades

In the early 1980s, the main site of CBG synthesis was established in the liver. Several groups subsequently showed that the protein was detected in other tissues, even within cells, although it was unclear whether this was local production or not. A hypothesis was then put forward regarding the potential internalization of circulating CBG by target cells. This hypothesis is based on the existence of a receptor capable of binding CBG to enable its endocytosis. In 1983, Strel’chyonok and Avvakumov were the first to suggest the presence of this receptor in the plasma membrane of human liver cells (211). Hryb and his colleagues confirmed this result on cell membranes from human prostate. Their study underlines the specificity of the binding of the receptor to CBG since albumin or transferrin are incapable of shifting the binding equilibrium. They also demonstrate that this binding varies depending on time and temperature (212). The presence of this receptor has also been demonstrated in the endometrium (213). It should be noted that *in vitro* studies show that only 2% to 5% of CBG bind to its receptor (212, 214). The binding of CBG to its receptor also appears to be dependent on prior binding of CBG to one of its ligands. Data in the literature are contradictory on this subject. Strel’chyonok and Avvakumov show that CBG binds to its receptor only if it is already bound to a GC, while Maitra and colleagues observe that binding CBG to a GC prevents it from binding to its receptor (214, 215). Since CBG is thermosensitive, these differences may be explained by differences in protocol, the first study having been carried out at 4 °C while the second was at 37 °C.

Three decades after these studies, a molecular characterization of such a receptor is still needed and one may question the reliability of the data. Only *in vitro* studies have indirectly shown its existence and a partial characterization has been carried out in rats (214). The few studies of this receptor have, however, led to the emergence of two hypotheses on its role. The first indicates that CBG, bound to its ligand, would be internalized in the cell and would thus allow finer control of the distribution of its ligands in its target tissues. This hypothesis thus gives CBG an active role in the addressing of its ligands. The second hypothesis considers CBG as a pro-hormone with intrinsic activity, the binding to its receptor allowing the activation of a second messenger. Nakhla and his colleagues demonstrate, in fact, that the binding of CBG to the plasma membrane leads to an activation of adenylate cyclase and an increase in cAMP in the cell (216). CBG would thus induce rapid intracellular signaling cascades and allow glucocorticoids to act rapidly in a non-transcriptional manner.

## 5 Physiopathologies in human

### 5.1 Endocrine diseases

Thyroid diseases can influence corticosteroid metabolism, with hyperthyroidism being associated with an increase in their catabolism (217). Consequences have also been observed on CBG, the plasma level of which decreases in patients with hyperthyroidism (218). The opposite has also been observed in patients with hypothyroidism (219). After treatment allowing a return to euthyroidism in these two types of patients, the CBG concentration normalizes (218–220). Hormones thus appear to negatively regulate CBG expression since *in vitro* studies show that long exposure of HepG2 cells to triiodothyronine causes it to decrease (221).

Changes in plasma CBG levels have also been observed in pathologies related to altered cortisol levels. Thus, in patients suffering from Cushing’s disease, a disease defined by chronic hypercortisolism, a decrease in plasma CBG levels is observed, accompanied by an increase in free cortisol levels (222, 223). The decrease in CBG concentration is associated with a decrease in binding capacity of up to 40% (63). The decrease in CBG could be the consequence of the regulation of its gene expression by cortisol. However, CBG levels appear to be normal in patients with Addison’s disease, a disease characterized in particular by a defect in cortisol secretion (222, 224). It should be noted that this disease is also associated with a defect in mineralocorticoid secretion. Therefore, the regulation of CBG in this context cannot be explained solely through the prism of cortisol levels.

CBG levels also appear to be decreased during obesity (225). As previously seen, some polymorphisms in the SERPINA6 gene have been associated with decreased plasma CBG concentrations and certain obesity-related parameters, such as insulin resistance (156). However, several studies have produced conflicting results on plasma CBG levels and insulin sensitivity (159, 225, 226). *In vitro*, in HepG2 cells, insulin is able to decrease CBG secretion and mRNA production (227). *In vivo*, in lean subjects, insulin injection causes a brief decrease in plasma CBG, but not in obese subjects (225). The regulation of plasma CBG in the context of obesity therefore appears to be more complex since obesity is also a chronic inflammatory pathology, a type of pathology that also causes variations in CBG levels.

### 5.2 Pro-inflammatory diseases

Given the role of CBG in inflammation, several studies have focused on its regulation in a pathological and inflammatory context. Savu and collaborators show a depletion of cortisol binding activity by CBG in patients with septic shock, reflecting a decrease in plasma levels (228). Pugeat and colleagues then directly demonstrate a drastic decrease in plasma CBG concentration in patients with septic shock (229). Monitoring a patient 9 days after shock reveals a progressive return to normal of CBG levels. However, the decrease in CBG was not observed for toxic, hemorrhagic or cardiogenic shock. The decrease in CBG during septic shock has been attributed to the regulation of CBG by inflammation, with interleukin-6 (IL-6), a central cytokine in inflammation, being able to inhibit its expression and secretion. Plasma IL-6 levels have indeed been correlated with plasma CBG levels in these patients: the higher the IL-6 level, the lower the CBG level (230). CBG levels have more recently been directly correlated with shock severity: patients who did not survive shock had the lowest plasma CBG levels (231). These findings have highlighted the importance of CBG in septic shock, with finally CBG deficiency independently associated with mortality (232).This same decrease in CBG was found in patients with burns or necrotizing pancreatitis (233, 234). In burn patients, this decrease was also correlated with an increase in IL-6. All the data in the literature on diseases with an inflammatory component suggest that CBG could be used as a biomarker of the degree of inflammation. It should be noted that, in the case of pancreatitis, the decrease in CBG in the first 48 hours has even been proposed as a predictive marker of future infection, with a positive predictive value of 100% and a negative predictive value of 87.5% (234). Concerning rheumatoid arthritis, contradictory data exist. Patients with rheumatoid arthritis either do not present change in plasma CBG levels compared to healthy controls (235), or show higher total and high-affinity CBG, reflecting reduced CBG cleavage in this pathology (236). Reduced plasma levels of CBG have been also recently reported in patients with coronavirus-19 disease (237).

To better understand the regulation of CBG in the inflammatory context of diseases, *in vitro* studies were conducted on the regulation of CBG in inflammatory conditions. They tend to show an inhibitory effect of inflammation on CBG expression. In 1993, Bartalena and colleagues first showed that IL-6 decreased CBG synthesis by HepG2 (238). The authors observed a decrease in CBG secretion in the media as well as a decrease in mRNA, in a dose- and time-dependent manner. However, they did not observe variations in the transcription rate and then hypothesized that IL-6 would decrease the stability of CBG mRNA. This *in vitro* effect of IL-6 on CBG expression was confirmed a few years later by another team who showed a decrease in CBG of up to 30% to 40% (64). Interestingly, this decrease is even more significant when the cells are treated with a combination of IL-6 and dexamethasone. The combination of these two molecules increases the expression of a subunit of the IL-6 receptor and thus potentiates the effects of IL-6. These two articles are at the origin of the classification of CBG as a negative acute phase protein in humans, a classification already carried out in rats in 1980 (239). This makes it possible to measure the severity of inflammation by the extent of the decrease in CBG: CBG could therefore be a biomarker of inflammation (240). The effect of IL-6 has also been studied *in vivo* on healthy subjects. A high-dose injection of IL-6 (3.0 μg/kg) decreases plasma CBG levels, which only return to normal after 7 days (241). However, Emptoz-Bonneton and colleagues also show that another pro-inflammatory molecule, IL-1β, causes an increase in CBG secretion at the same time as its decrease in mRNA (64). IL-1β would thus act post-transcriptionally and/or directly on the CBG secretion mechanism. Inflammation could therefore act on CBG according to different mechanisms that remain to be elucidated.

### 5.3 Surgical field and procedures

Tinnikov and colleagues were the first to show, in children undergoing cardiac surgery, that plasma CBG levels decreased by half during the procedure, while cortisol levels increased (199). CBG levels also remained low the day following surgery. This decrease has been confirmed in other cohorts (200). Roth-Isigkeit and colleagues, however, noted a decrease in hematocrit percentage during surgery and over the following two days (201). Correction by hemodilution shows that the CBG level is only slightly decreased, and this only on the day of surgery. The corrected total and free cortisol concentration is still increased for several postoperative days. Their study thus shows that, in this context, cortisol secretion seems to be the main determinant of the free cortisol level, the CBG level not being altered.

### 5.4 Hepatic diseases

Since CBG is mainly produced by the liver, studies have focused on its plasma level in the context of liver diseases. Shortly after its discovery, Doe and colleagues showed that patients suffering from cirrhosis had a lowered plasma CBG concentration (242). Several studies then validated this result on different types of cirrhosis including cirrhosis caused by biliary atresia, hepatitis B or C virus, and autoimmune disease (55, 243). The decrease in plasma CBG concentration seems to be correlated with the severity of cirrhosis: the more pronounced the liver damage, the more the CBG level falls (244). Interestingly, another study investigating new markers of liver fibrosis identified CBG as a potential biomarker, with its plasma level also decreasing with increasing severity of the disease (245). The decrease in plasma CBG has been explained by a probable decrease in synthesis at the hepatic level, although no study has been able to demonstrate this to date.

### 5.5 The specific cystic fibrosis status

Cystic fibrosis (CF) is a genetic disease with both pro-inflammatory profile and, for some patients, liver condition; some CF patients requiring liver transplant. We expected that, in CF patients suffering from liver hepatic disease (cirrhosis), the levels of both liver and serum CBG would be decreased, as a pioneer study reported a slight decrease in plasma CBG capacity in CF patients with low liver condition (246). We obtained exactly the opposite with significant increase of both transcripts and protein in cirrhotic liver from CF patients compared to healthy donors and cirrhotic non-CF patients (207). It is unlikely that the hepatic increase in CBG transcripts and protein is a direct consequence of a CFTR channel dysfunction within the cell, as CBG is produced by hepatocytes (247) when CFTR is exclusively expressed by cholangiocytes (248). The cause is rather the specific microenvironment of CF liver with inflammation due to toxic bile acids accumulation (249). In our study, the plasma levels of CBG were unchanged among patients analyzed (207). On the other hand, this could be due to a hepatic retention, but the plasma levels of other proteins produced and secreted by the liver are not disrupted in CF patients (250). On the other hand, an increased cleavage of CBG in plasma can be considered as we also observed an increase of elastase/α-1-antitrypsin complex in the plasma of CF patients (207). When one knows that elastase is sustainably released in CF patients due to an exacerbated neutrophilic activity (187) and to the early colonization of patients by *Pseudomonas aeruginosa* (188), this enzyme could target CBG and cleave it, leading to its irreversible inactivation (187, 192).

## 6 Conclusion

The aim of this review was to give a state-of-the-art on CBG in vertebrates including binding, addressing, and reservoir. Each function can be understood individually but CBG is likely to perform all of these functions simultaneously. For each of the functions described, the debate is still opened, including the ability of extrahepatic CBG to be released in the circulation. Our main objective was not to conclude on these functions as many issues need further studies. Ultimately, we suggest that CBG could be considered as a perfect “pleiotropic” partner for the pleiotropic glucocorticoid.

In medical field, CBG can represent an important serum marker, sometimes associated to life prognostic as in the probability of survival from septic shock. Its regulation seems to be closely dependent on pro-inflammatory factors in the patient. Interestingly, several studies show that the main GC prescribed for CF patients (prednisone and prednisolone) have very poor to no affinity for CBG (251, 252). This lack of affinity leads to the use of high doses to obtain enough GC at the inflammatory site. These GC, with high hydrophobic profile, are not limited exclusively to this site, as if they were binding to CBG, but they act within the whole body with metabolic, immune, and developmental side effects (253). Using a GC with a high affinity for CBG could be a way of reducing the side effects, as already suggested in the literature (190, 254).

The also called transcortin, a name given 50 years ago, is not anymore a stranger, thanks to the community of scientists who developed research to better understand CBG. But, in the complex context of GC regulation, CBG still needs to take its right place among HPA/HPI axis, glucocorticoid receptors or 11 β-HSD activity, that control GC delivery and efficacy. CBG, 50 years after its discovery, sometimes remains a new guest for an old ceremony.
